# Letter to the editor regarding the editorial of issue 2/2022 “Taking responsibility” by Sigrid Harendza

**DOI:** 10.3205/zma001581

**Published:** 2022-11-15

**Authors:** Udo Obertacke, Elisabeth Narciss, Harald Fritz

**Affiliations:** 1 Universitätsmedizin Mannheim, Ruprecht-Karls-Universität Heidelberg, Klinik für Unfallchirurgie, OUZ, Mannheim, Germany; 2 Universitätsmedizin Mannheim, Ruprecht-Karls-Universität Heidelberg, Studiendekanat Kompetenzzentrum PJ, Mannheim, Germany; 3 Universitätsmedizin Mannheim, Ruprecht-Karls-Universität Heidelberg, Geschäftsbereich Studium und Lehrentwicklung, Mannheim, Germany

## Letter to the editor

Dear Editors,

in the editorial “Taking responsibility” [1], it is argued that students be familiarized with the need to take responsibility and given opportunity to practice taking responsibility. So far, so disturbing.

In the age group to which our students belong, a wide variety of different professionals go about doing their work. Who taught these professionals “responsibility”? Do we need special tutoring on responsibility in our society? Is responsibility to be expected of medical students only once they turn 25 and is the university “responsible” for their doing so?

How long should the brightest graduates in Germany go on being sheltered before professional work is simply expected of them without excuse? And it goes without saying that this includes, at the least, taking responsibility for their own proficiency in diagnosis, therapy and communication, for their own actions or lack of action, etc.

Of course, it is lamentable that studying medicine is only somewhat suited to develop one's personality. But what is a master craftsman to say when asked about how apprentices learn responsibility? The role model that is often striven for has little influence here since we increasingly see that although role models are accepted, they are not imitated: “Someone else will surely come and do the work.”

We should not reinforce the tendency toward infantilization, to which our students are all too happy to succumb, by counteracting obvious deficiencies – among them immaturity – in academic course formats. This infantilization is visible on a daily basis and is also eagerly instrumentalized in ways ranging from claims that “no one showed me that” to positively regressive behavior, even in the final practical year of undergraduate medical education.

Taking responsibility is a question of character and whoever is unable to do so should not be licensed to practice medicine, at least not for a long time.

This could certainly be determined (assessed?), at the latest during the final year of medical study, through continual observation in the clinical setting.

We know that solutions are not simple to implement. But yet more “sheltering” of adults in the form of micro-managed rules and guidelines and learning objectives is also not a solution, as is proposed in the current discussion of the new medical licensing regulations (ÄApprO).

One observation that surprised us is that this kind of infantilization is much less present in students who received patient-centered training prior to studying medicine. An increase in the quality and quantity of *(ongoing) contact with patients* (starting at the beginning of medical study and not just in 45-minute units) could lead a better definition of and identification with the physician’s role and an improvement in the willingness to take responsibility.

## Competing interests

The authors declare that they have no competing interests. 
